# 抗血管生成药物联合EGFR-TKI治疗*EGFR*突变晚期非小细胞肺癌的研究进展

**DOI:** 10.3779/j.issn.1009-3419.2022.101.41

**Published:** 2022-08-20

**Authors:** 博文 李, 剑超 薛, 亚东 王, 志诚 黄, 乃新 梁, 单青 李

**Affiliations:** 100730 北京，中国医学科学院，北京协和医学院，北京协和医院胸外科 Department of Thoracic Surgery, Peking Union Medical College Hospital, Chinese Academy of Medical Sciences (CAMS) and Peking Union Medical College (PUMC), Beijing 100730, China

**Keywords:** 肺肿瘤, 表皮生长因子受体, 抗血管生成药物, 联合治疗, Lung neoplasms, Epidermal growth factor receptor, Antiangiogenic drugs, Combined modality therapy

## Abstract

肺癌是最常见、致死率最高的肿瘤性疾病之一，使用表皮生长因子受体酪氨酸激酶抑制剂（epidermal growth factor receptor tyrosine kinase inhibitor, EGRF-TKI）已经成为*EGFR*突变晚期非小细胞肺癌（non-small cell lung cancer, NSCLC）的标准治疗方式。然而耐药事件的发生往往是不可避免的。EGFR-TKI联合抗血管生成药物（angiogenesis inhibitor）是目前一种正在探索的延缓耐药事件发生的治疗方案，被称为“A+T治疗”。多项临床试验已经证明A+T治疗相较于单药可以延长患者的无进展生存期（progression free survival, PFS）。然而，基于不同EGFR-TKI的A+T治疗相对于EGFR-TKI单药治疗所带来的获益程度、安全性和探索前景仍不明确。因此，我们回顾了第一、二、三代EGFR-TKI联合抗血管生成药物治疗的相关文献，总结了A+T治疗模式的增益机制、获益程度、安全性以及最佳的目标人群。

## Abstract (en)

Lung cancer is one of the leading causes of cancer-related morbidity and mortality. Epidermal growth factor receptor tyrosine kinase inhibitor (EGFR-TKI) have become the standard treatment for *EGFR*-mutated advanced non-small cell lung cancer (NSCLC). Unfortunately, drug resistance is inevitable in most cases. EGFR-TKI combined with angiogenesis inhibitors is a treatment scheme being explored to delay the therapeutic resistance, which is called "A+T treatment". Several clinical trials have demonstrated that the A+T treatment can improve the progression free survival (PFS) of the NSCLC patients. However, compared to EGFR-TKI monotherapy, the benefits of the A+T treatment based on different EGFR-TKIs, as well as its safety and exploration prospects are still unclear. Therefore, we reviewed the literature related to all three generations EGFR-TKIs combined with angiogenesis inhibitors, and summarized the mechanism, benefit, safety, optimal target population of A+T treatment.

肺癌是最常见的肿瘤性疾病之一，并且是癌症相关死亡的首要原因^[1]^。非小细胞肺癌（non-small cell lung cancer, NSCLC）占肺癌的85%，而且相当一部分患者在发现病灶时已经进入了晚期^[2]^。NSCLC病理类型复杂、驱动基因多样，具有很强的疾病异质性^[3]^。表皮生长因子受体（epidermal growth factor receptor, *EGFR*）基因是NSCLC最常见的突变驱动基因，40%-60%的亚裔肺腺癌患者及10%-20%的高加索裔肺腺癌患者携带*EGFR*突变^[4]^，外显子19缺失（19 exon deletion, Ex19del）和外显子21 L858R突变（L858R mutation in 21 exon, Ex21L858R）占其中的90%^[5]^。EGFR酪氨酸激酶抑制剂（EGFR tyrosine kinase inhibitor, EGFR-TKI）的发现有效延长了*EGFR*敏感突变（主要为Ex19del或Ex21L858R）晚期NSCLC患者的缓解期和生存期^[6]^。尽管目前已经有三代EGFT-TKI药物上市，然而无论接受哪一代药物治疗，患者都不可避免地会面对耐药事件的发生^[7]^。在*EGFR*突变晚期NSCLC患者的一线治疗中，第一代EGFR-TKI（如：吉非替尼、厄洛替尼、埃克替尼）、第二代EGFR-TKI（如：阿法替尼、达可替尼）和第三代EGFR-TKI（如：奥希替尼）的中位无进展生存期（median progression-free survival, mPFS）分别为8个月-13个月、11个月-15个月和18.9个月；而中位总生存期（median overall survival, mOS）则分别为17个月-34个月、28个月-34个月和38.6个月^[8-14]^。延缓肿瘤对EGFR-TKI耐药成为亟待解决的问题。

早在化疗时代，就有研究^[15]^提出贝伐珠单抗（Bevacizumab）联合化疗相比于单纯化疗可以显著延长NSCLC患者的缓解期和OS。因此，阻断血管内皮生长因子（vascular endothelial growth factor, VEGF）通路的抗血管生成药物（angiogenesis inhibitor）联合EGFR-TKI成为目前临床试验的重点探索方向之一。最初，以贝伐珠单抗联合厄洛替尼（Erlotinib）为代表的联合方案被称为“A+T模式”（Avastin+Tarceva）。随着EGFR-TKI种类和代数的增加，这一概念也逐渐升级为更广义的“A+T”（Angiogenesis inhibitor+Tyrosine kinase inhibitor）。尽管相关研究已经大量开展，但目前对A+T模式协同增效的机制、A+T模式相较于EGFR-TKI单药治疗的额外获益程度、不良反应的增加以及耐药机制的变化尚未有系统化的整理。本文将梳理上述内容，同时通过整理相关亚组分析结果探寻A+T模式潜在的最佳目标人群特点，为未来A+T模式的研究提供方向。

## 1 抗血管生成药物与EGFR-TKI联合作用协同增效的机制

### 1.1 分子层面

具有获得性EGFR-TKI耐药性的肿瘤可能表现出VEGF表达的显著上调^[16]^，暗示增强VEGF通路信号是肿瘤对EGRF-TKI耐药的潜在机制之一。这种耐药机制可能基于肿瘤和肿瘤相关内皮细胞中广泛存在着自分泌或旁分泌EGFR与VEGF通路的交互作用。由EGFR介导的信号可以通过MAPK和PI3K级联途径、至少3种转录因子（STAT3、Sp1和HIFs）上调VEGF的表达^[17]^，还可以导致许多其他刺激血管生成的小分子物质合成增加，如白细胞介素（interleukin, IL）-8、血管生成素-1、血管生成素-2和碱性成纤维细胞生长因子等^[18]^。阻断VEGF信号通路也可以显著降低EGFR信号通路的磷酸化水平，使EGFR自分泌信号下降^[19]^。Bozec等^[20]^在体外实验中提出“联合比”的概念，提示抗血管生成药物与EGFR-TKI组合的抗肿瘤效果超过了单纯的累加效应，暗示二者具有协同抗肿瘤活性。

### 1.2 细胞及组织层面

贝伐珠单抗在异种移植瘤模型中可以诱导肿瘤微血管密度显著、快速、渐进性降低，同时提高血管成熟指数，从而诱导肿瘤血管通透性下降，降低肿瘤间质液体压力，增加肿瘤内灌注，改善EGFR-TKI的肿瘤渗透性。但值得注意的是，这种效果似乎是短暂和一过性的，这可能归因于治疗后期肿瘤血管数量的减少^[21]^。体外实验^[22]^也证实了联用贝伐珠单抗可以增加肿瘤内部厄洛替尼的浓度。

### 1.3 器官及系统层面

S100A9阳性的骨髓源性抑制细胞（myeloid-derived suppressor cells, MDSCs）可以被招募到脑组织中，构造出适合肿瘤种植的微环境，在脑转移瘤的形成中起着重要作用。贝伐珠单抗可以减少NSCLC患者外周血中S100A9阳性MDSCs，有效延缓脑转移^[23]^。

## 2 第一代EGFR-TKI联合抗血管生成药物

### 2.1 有效性

在BeTa研究的亚组分析结果显示亚裔、*EGFR*突变患者有从A+T模式中获益的趋势后^[24]^，一系列关于厄洛替尼+贝伐珠单抗 *vs* 厄洛替尼单药一线治疗*EGFR*突变晚期NSCLC的临床试验迅速开展。JO25567研究（Ⅱ期试验）和NEJ026（Ⅲ期试验）在日本先后进行，报道了厄洛替尼联合贝伐珠单抗相较于厄洛替尼单药一线治疗*EGFR*敏感突变晚期NSCLC，PFS显著获益^[25, 26]^，但OS没有显著差异的现象^[27, 28]^。例如在NEJ026研究中，A+T组对比厄洛替尼单药组：mPFS（16.9个月 *vs* 13.3个月，HR=0.605，95%CI：0.417-0.877，*P*=0.016）有显著统计学差异^[26]^，mOS（50.7个月 *vs* 46.2个月，HR=1.007，95%CI：0.681-1.490，*P*=0.97）无显著统计学差异^[28]^。

在中国开展的Ⅲ期临床研究CTONG1509^[29]^也得到了同样的结论，同时还提出两组之间的客观缓解率（objective response rate, ORR）（86.8% *vs* 84.7%, *P*=0.624）和疾病控制率（disease control rate, DCR）（96.1% *vs* 96.7%, *P* > 0.999）也没有明显差异，这可能是因为EGFR-TKI单药本身就能取得非常好的初期消退肿瘤的效果。

不同的A+T组合形式也在探索中。全球多中心、双盲Ⅲ期临床试验RELAY研究^[30]^探索了厄洛替尼联合雷莫芦单抗（Ramucirumab），同样验证了A+T方案带来了PFS获益，但没有转化为OS获益（从已公布的数据来看）；除东亚人群外，其他地区人群同样有PFS获益的趋势，尽管因为样本量较小（113/449; 25%）而处于显著水平的边界（HR=0.61, 95%CI: 0.36-1.01）。在CTONG1706研究^[31]^中，吉非替尼（Gefitinib）联合阿帕替尼（Apatinib）相较于吉非替尼单药也在PFS上显著获益（13.7个月 *vs* 10.2个月，HR=0.71，95%CI：0.54-0.95，*P*=0.018, 9）。吉非替尼+呋喹替尼（Fruquintinib）的单臂试验^[32]^、埃克替尼（Icotinib）+贝伐珠单抗的回顾性研究^[33]^也分别取得了14.7个月、18.0个月的mPFS。因此从目前的数据来看，基于第一代EGFR-TKI的不同A+T组合都表现为PFS显著获益而OS缺乏显著获益（表 1）。

**表 1:** A+T治疗与EGFR-TKI单药治疗的获益比较 Benefit of A+T treatment compared with that of EGFR-TKI monotherapy

Study	A+T (*n*)	T (*n*)	mPFS (mon)	PFS HR (95%CI), *P*	mOS (mon)	OS HR (95%CI), *P*
Takashi Seto, 2014 (RCT)	Erlotinib+Bevacizumab (75)	Erlotinib (77)	16.0 *vs* 9.7	0.54 (0.36-0.79) *P*=0.002	47.0 *vs* 47.4	0.81 (0.53-1.23) *P*=0.327
Haruhiro Saito, 2019 (RCT)	Erlotinib+Bevacizumab (112)	Erlotinib (112)	16.9 *vs* 13.3	0.61 (0.42-0.88) *P*=0.016	50.7 *vs* 46.2	1.01 (0.68-1.49) *P*=0.970
Qing Zhou, 2021 (RCT)	Erlotinib+Bevacizumab (157)	Erlotinib (154)	17.9 *vs* 11.2	0.55 (0.41-0.73) *P* < 0.001	36.2 *vs* 31.6	0.92 (0.69-1.23) *P*=0.581
Kazuhiko Nakagawa, 2019 (RCT)	Erlotinib+Ramucirumab (224)	Erlotinib (225)	19.4 *vs* 12.4	0.59 (0.46-0.76) *P* < 0.001	NA	NA NA
Hongyun Zhao, 2021 (RCT)	Gefitinib+Apatinib (157)	Gefitinib (156)	13.7 *vs* 10.2	0.71 (0.54-0.95) *P*=0.019	NA	NA NA
Zhansheng Jiang, 2021 (Retrospective study)	Icotinib+Bevacizumab (30)	Icotinib (60)	18.0 *vs* 11.0	0.54 (0.31-0.92) *P*=0.029	NA	NA NA
A+T: EGFR tyrosine kinase inhibitor combined with angiogenesis inhibitor; EGFR-TKI: epidermal growth factor receptor tyrosine kinase inhibitor; T: EGFR-TKI; mPFS: median progression-free survival; mOS: median overall survival; RCT: randomized controlled trial; NA: not available.						

### 2.2 安全性

联合用药也会导致不良事件（adverse events, AEs）的增加，但这种增加似乎是两种药物AEs的简单“叠加”，不会出现AEs之间的协同促进以及新的AEs。EGFR-TKI相关的AEs主要有皮肤相关反应（如：皮疹、痤疮、皮肤干燥和甲沟炎等）、腹泻、转氨酶升高、间质性肺疾病（interstitial lung disease, ILD）等^[34]^；而抗血管生成药物（以贝伐珠单抗为例）相关AEs则包括出血、胃肠道穿孔、高血压、动脉血栓栓塞、蛋白尿/肾病综合征等^[35]^。除消化道症状外，二者几乎没有重合部分。Chen等^[36]^进行的*meta*分析显示，在比较全等级AEs时，A+T模式的出血（RR=2.62, 95%CI: 1.22-5.65）、高血压（RR=2.79, 95%CI: 1.64-4.74）、蛋白尿（RR=4.08, 95%CI: 1.47-11.37）、水肿（RR=5.09, 95%CI: 2.65-9.78）和脱发（RR=1.61, 95%CI: 1.02-2.54）发生率显著更高；在≥3级AEs中腹泻（RR=4.82, 95%CI: 1.74-13.32）、高血压（RR=3.95, 95%CI: 2.47-6.31）和蛋白尿（RR=12.70, 95%CI: 2.43-66.40）发生率显著更高。其中，出血、高血压和蛋白尿均是贝伐珠单抗的常见AEs。除≥3级腹泻外，联用抗血管生成药物并没有显著增加EGFR-TKI相关的AEs发生率。在A+T模式中没有出现与EGFR-TKI和抗血管生成药物均无关的新增AEs。几种不同A+T组合方式的≥3级AEs（高血压、蛋白尿、腹泻）发生率相差不大（表 2）。ILD是EGFR-TKI治疗中可能发生的AEs，但在A+T组中观察到的ILD发生率似乎更低（尽管没有达到统计学显著水平）^[26, 37]^，这可能归因于抗血管生成药物对肺组织潜在的保护效应^[38]^。

**表 2:** 几种A+T组合方案中发生一些不良事件的患者比例 Proportion of patients with some adverse events in several A+T combination regimens

Study	A+T (*n*)	Hypertension (All grades)/brhypertension (Grade≥3)	Proteinuria (All grades)/proteinuria (Grade≥3)	Diarrhea (Grade≥3)
Haruhiro Saito, 2019 (RCT)	Erlotinib+Bevacizumab (112)	52 (46.4%) 26 (23.2%)	36 (32.1%) 8 (7.1%)	6 (5.4%)
Qing Zhou, 2021 (RCT)	Erlotinib+Bevacizumab (157)	76 (48.4%) 29 (18.5%)	95 (60.5%) 11 (7.0%)	4 (2.5%)
Kazuhiko Nakagawa, 2019 (RCT)	Erlotinib+Ramucirumab (221)	100 (45.2%) 52 (23.5%)	75 (33.9%) 6 (2.7%)	16 (7.2%)
Eiki Ichihara, 2015 (Single arm study)	Gefitinib+Bevacizumab (41)	28 (68.3%) 7 (17.1%)	23 (56.1%) 3 (7.3%)	1 (2.4%)
Hongyun Zhao, 2021 (RCT)	Gefitinib+Apatinib (157)	107 (68.2%) 73 (46.5%)	89 (56.7%) 28 (17.8%)	14 (8.9%)
Zhansheng Jiang, 2021 (Retrospective study)	Icotinib+Bevacizumab (30)	24 (80.0%) 3 (10.0%)	18 (60.0%) 3 (10.0%)	2 (6.6%)
The proportion of patients with some AEs (hypertension and proteinuria) and grade≥3 AEs (hypertension, proteinuria and diarrhea) in several A+T combination regimens. In each study, data about all grade AEs are listed in the upper row, while data about grade≥3 AEs are listed in the lower row.				

在A+T组中，厄洛替尼的使用时间并不低于单药组，在治疗中厄洛替尼的停药率也没有因为联用抗血管生成药物而明显增加^[26, 29, 37]^。A+T模式的AEs很大程度上可以通过剂量调整和支持疗法来控制^[37]^。

### 2.3 亚组分析

在第一代EGFR-TKI联合抗血管药物的探索中，预设的各个亚组（按照性别、年龄、吸烟状态、*EGFR*突变亚型、有无脑转移、有无胸腔积液等分层）基本上都有从A+T模式中额外获益的趋势^[25, 26, 29]^。

#### 2.3.1 *TP53*共突变患者

*EGFR*突变晚期NSCLC患者合并*TP53*突变（54.6%-64.6%）十分常见，而且存在*TP53*共突变的患者预后较差，使用EGFR-TKI的效果不佳^[39]^。*TP53*突变上调VEGF-A和VEGFR2表达水平^[40]^，当肿瘤存在*TP53*突变时，使用贝伐珠单抗疗效更佳且能获得更长的PFS^[41]^。在EGFR-TKI治疗肿瘤的过程中突变型*TP53*阳性率上升，意味着其可能是一种潜在的耐药机制^[42]^。这些都为A+T模式的应用提供了理论基础。

CTONG1706研究^[31]^在吉非替尼治疗中联用阿帕替尼，相对于吉非替尼单药使*TP53*共突变患者的进展风险降低44%（*TP53*外显子8突变患者进展风险降低76%），而仅使TP53野生型患者的进展风险降低8%，提示A+T带来的PFS获益可能主要集中在*TP53*突变亚组。在RELAY研究^[43]^中，联用雷莫芦单抗后，*TP53*共突变患者在Ex19del和Ex21L858R亚组中均有显著PFS获益，而TP53野生型患者中仅L858R患者有获益趋势（表 3）。尚未完全公布数据的ALTER-L004研究^[44]^也显示携带伴随突变的患者使用埃克替尼联合安罗替尼（Anlotinib）治疗的ORR达80.95%，而在无伴随突变的患者中ORR仅为40%。除*TP53*突变外，其他抑癌基因共突变或其他伴随突变是否能从A+T模式中更大程度获益尚待探索。

**表 3:** A+T治疗与EGFR-TKI在*TP53*突变型或TP53野生型患者中的疗效比较 A+T treatment versus EGFR-TKI in *TP53*-mutated or TP53 wild-type patients

Study	Subgroup	A+T (*n*)	T (*n*)	mPFS (mon)	PFS HR (95%CI)
Kazuhiko Nakagawa, 2019 (RCT)	Overall	Erlotinib+Ramucirumab (224)	Erlotinib (225)	19.4 *vs* 12.4	0.59 (0.46-0.76)
	Ex19del mTP53	37	50	18.0 *vs* 9.9	0.50 (0.29-0.85)
	Ex21L858R mTP53	37	41	14.7 *vs* 10.8	0.56 (0.34-0.95)
	Ex19del wTP53	65	52	20.6 *vs* 19.4	1.00 (0.62-1.61)
	Ex21L858R wTP53	51	51	20.8 *vs* 13.8	0.60 (0.35-1.02)
Hongyun Zhao, 2021 (RCT)	Overall	Gefitinib+Apatinib (157)	Gefitinib (156)	13.7 *vs* 10.2	0.71 (0.54-0.95)
	mTP53	30	40	12.5 *vs* 10.1	0.56 (0.31-1.01)
	wTP53	43	32	15.6 *vs* 12.0	0.92 (0.50-1.67)
mTP53: mutant-type TP53; wTP53: wild-type TP53.					

#### 2.3.2 脑转移患者

*EGFR*突变晚期NSCLC患者中脑转移发生率可以达到50%^[45]^。CTONG1509研究中脑转移患者的mPFS达到17.9个月（95%CI: 15.2-20.7）^[29]^，在数值上超过了FLAURA研究中奥希替尼（Osimertinib）实现的15.2个月（95%CI: 12.1-21.4）^[13]^，与该研究中无脑转移亚组的mPFS相似（17.9个月，95%CI: 13.8-20.8），提示脑转移患者联用抗血管生成药物降低进展风险的程度高于无脑转移患者（PFS HR: 0.48 *vs* 0.57），降低死亡风险的程度也更大（OS HR: 0.62 *vs* 1.09）^[29]^。Wang等^[46]^进行的回顾性研究也证明，第一代EGFR-TKI联合贝伐珠单抗相对于EGFR-TKI单药或EGFR-TKI联合化疗，可以提高对颅内病变的疗效（颅内ORR：60.0% *vs* 17.9% *vs* 37.3%）；联合贝伐珠单抗的治疗方案可以显著延缓颅内病灶进展（颅内PFS：21.0个月 *vs* 11.3个月，*P*=0.007）。Feng等^[23]^提出在A+T组中脑转移作为首次进展部位的患者比例显著低于EGFR-TKI单药组（38.0% *vs* 71.0%, *P*=0.03），至发生颅内进展的中位时间也显著延长（49.1个月 *vs* 12.9个月，*P*=0.002），提示了抗血管生成药物的联用对预防脑转移的较好效果。然而，许多研究出于安全性考虑（脑出血）不纳入脑转移患者或仅纳入无症状、稳定的脑转移患者。部分研究也没有发现脑转移患者获益程度更大，因此上述结论仍需要进一步验证。此外，尽管中枢神经系统出血是脑转移患者联用贝伐珠单抗最受关注的并发症之一，但Besse等^[47]^已经证明在肿瘤脑转移患者中使用贝伐珠单抗并不会显著增加脑出血的风险。

#### 2.3.3 Ex21L858R患者相比于Ex19del患者能否从A+T模式中获益更多尚存在争议

在体外试验^[48]^中，第一代EGFR-TKI抑制Ex19del细胞*EGFR*、*Akt*和*Erk*磷酸化的程度高于Ex21L858R细胞；一项*meta*分析^[49]^也证实Ex19del患者使用第一代EGFR-TKI的疗效优于Ex21L858R患者。然而在CTONG1509、NEJ026中，A+T模式更大程度降低了Ex21L858R患者的进展风险（PFS HR_Ex21L858R_ < PFS HR_Ex19del_），甚至消除了EGFR-TKI在两种突变亚型中的疗效差异。例如在CTONG1509研究^[29]^中，A+T模式使Ex19del患者的mPFS从12.5个月提升到17.7个月（HR=0.62, 95%CI: 0.42-0.93），而使Ex21L858R患者的mPFS从9.7个月提升到19.5个月（HR=0.50, 95%CI: 0.32-0.77）。

尽管上述研究在Ex21L858R患者中取得了十分耀眼的成绩，但一些研究如CTONG1706的结果却提示Ex19del患者从A+T模式中获益更多（表 4）。在近期公布的、同样为厄洛替尼联合贝伐珠单抗的BEVERLY研究^[50]^中，也提示在PFS方面Ex19del患者从联合方案中得到了更多的额外获益；但有趣的是，在OS方面Ex21L858R患者额外获益的程度却超过了Ex19del患者。基于上述结果，Ex21L858R患者是否更适合A+T模式尚存在争议。

**表 4:** A+T治疗与EGFR-TKI在Ex19del或Ex21L858R患者中的疗效比较 A+T treatment versus EGFR-TKI in patients with Ex19del or Ex21L858R

Study	Subgroup	A+T (*n*)	T (*n*)	mPFS (mon)	PFS HR (95%CI)
Haruhiro Saito, 2019 (RCT)	Overall	Erlotinib+Bevacizumab (112)	Erlotinib (112)	16.9 *vs* 13.3	0.61 (0.42-0.88)
	Ex19del	56	55	16.6 *vs* 12.4	0.69 (0.41-1.16)
	Ex21L858R	56	57	17.4 *vs* 13.7	0.57 (0.33-0.97)
Qing Zhou, 2021 (RCT)	Overall	Erlotinib+Bevacizumab (157)	Erlotinib (154)	17.9 *vs* 11.2	0.55 (0.41-0.73)
	Ex19del	82	79	17.7 *vs* 12.5	0.62 (0.42-0.93)
	Ex21L858R	75	75	19.5 *vs* 9.7	0.50 (0.32-0.77)
Kazuhiko Nakagawa, 2019 (RCT)	Overall	Erlotinib+Ramucirumab (224)	Erlotinib (225)	19.4 *vs* 12.4	0.59 (0.46-0.76)
	Ex19del	123	120	19.6 *vs* 12.5	0.65 (0.47-0.90)
	Ex21L858R	99	105	19.4 *vs* 11.2	0.62 (0.44-0.87)
Hongyun Zhao, 2021 (RCT)	Overall	Gefitinib+Apatinib (157)	Gefitinib (156)	13.7 *vs* 10.2	0.71 (0.54-0.95)
	Ex19del	81	83	14.1 *vs* 11.9	0.67 (0.45-0.99)
	Ex21L858R	74	73	11.9 *vs* 10.1	0.72 (0.48-1.09)
Zhansheng Jiang, 2021 (Retrospective study)	Overall	Icotinib+Bevacizumab (30)	Icotinib (60)	18.0 *vs* 11.0	0.54 (0.31-0.29)
	Ex19del	18	39	17.0 *vs* 12.0	0.81 (0.41-1.60)
	Ex21L858R	12	21	NA *vs* 11.0	0.53 (0.31-0.92)

### 2.4 继发T790M突变比例

第一/二代EGFR-TKI一线用药后的耐药机制对二线用药具有重要的指导意义。发生外显子20 T790M耐药突变的患者可以二线使用奥希替尼，因此A+T模式是否改变T790M突变比例是十分重要的问题。CTONG1509研究^[29]^对A+T组与厄洛替尼单药组中疾病进展（progressive disease, PD）的患者进行耐药原因分析，发现两组之间T790M突变比例并没有显著差异（33% *vs* 45%, *P*=0.187）。NEJ026和RELAY研究也得到同样的结论。但是，这些分析很大程度上是建立在对外周血中游离DNA（cell-free DNA, cfDNA）的分析，而非后续穿刺获得的肿瘤标本，因此可靠性仍待检验。尽管没有统计学上的差异，但多数研究中A+T组进展患者的T790M突变比例在数值上有更低的趋势，且结论源于小样本量的亚组分析，因此应谨慎解读。一项使用肿瘤石蜡包埋标本的回顾性研究^[42]^发现A+T组耐药患者T790M阳性率显著低于EGFR-TKI单药组（35.5% *vs* 51.5%, *P* < 0.001）。关于A+T模式耐药机制的探索仍需要基于肿瘤标本活检的大型Ⅲ期临床试验的验证。

## 3 第二代EGFR-TKI联合抗血管生成药物

仅有一些回顾性研究和单臂试验探索了第二代EGFR-TKI联用抗血管生成药物的可行性，这可能是考虑到第二代EGFR-TKI本身就会引起较多AEs^[51]^。

### 3.1 有效性

一项回顾性研究（*n*=405）^[52]^发现阿法替尼+贝伐珠单抗相较于阿法替尼单药一线治疗*EGFR*敏感突变晚期NSCLC患者在PFS（16.1个月 *vs* 15.0个月，*P*=0.500）和OS（32.1个月 *vs* 42.0个月，*P*=0.700）方面均没有显著获益，各亚组分析也没有阳性结果。另有一项单臂研究^[53]^提出阿法替尼（Afatinib）+贝伐珠单抗在既往未经过EGFR-TKI治疗的患者中实现了16.8个月的mPFS。此外，Hata等^[54]^采用阿法替尼+贝伐珠单抗联合方案，在既往对一代或二代TKI耐药的患者中取得了6.3个月的mPFS。

### 3.2 安全性

关于AEs的数据较少，一项单臂研究^[53]^指出日本人群使用贝伐珠单抗联合30 mg/d的阿法替尼耐受性较好，而联合40 mg/d（标准剂量）的阿法替尼则难以耐受。鉴于这些回顾性研究和单臂试验的结果并不支持联合治疗有额外的PFS或OS获益，且暗示标准剂量下的联合用药可能会造成难以接受的AEs发生率，继续探索第二代EGFR-TKI联合抗血管生成药物的意义可能不大。

### 3.3 继发T790M突变比例

Kuo等^[52]^开展的回顾性研究提出基于第二代EGFR-TKI的A+T模式相比于单药在耐药后T790M突变比例不会减少（56.3% *vs* 49.4%, *P*=0.794）。同前所述，该结论需要进一步验证。

## 4 第三代EGFR-TKI联合抗血管生成药物

由于第三代EGFR-TKI在临床上常用于既往第一/二代EGFR-TKI治疗后发生T790M耐药突变的患者，一些临床研究迅速开展，探究奥希替尼联合抗血管生成药物能否为发生T790M耐药突变患者带来更佳的临床结局。

### 4.1 有效性

Ⅱ期临床试验WJOG8715L^[55]^率先提示治疗既往第一/二代EGFR-TKI耐药且证实为T790M突变晚期肺腺癌患者时，奥希替尼+贝伐珠单抗（A+T组）相较于奥希替尼单药并不能延长PFS（9.4个月 *vs* 13.5个月，HR=1.44，95%CI：0.83-2.52，*P*=0.20）。这一结果引起了广泛讨论。另一项在相似入组条件下进行的全球多中心双盲Ⅱ期临床研究BOOSTER^[56]^也表明，奥希替尼+贝伐珠单抗模式下PFS（15.4个月 *vs* 12.3个月，HR=0.94，95%CI：0.66-1.33，*P*=0.71）、OS（24.0个月 *vs* 24.3个月，HR=1.03，95%CI：0.68-1.56，*P*=0.89）、ORR（55% *vs* 55%）、DCR（90% *vs* 82%, *P*=0.17）、响应持续时间（duration of response, DOR）（14.5个月 *vs* 16.6个月，*P*=0.36）、临床获益持续时间（duration of clinical benefit, DOCB）（14.5个月 *vs* 12.5个月，*P*=0.76）相较于奥希替尼单药均没有显著的提高。

奥希替尼联合其他抗血管生成药物的组合也没有取得阳性结果。Yu等^[57]^进行的Ⅰ期单臂试验在25例一线EGFR-TKI治疗耐药的T790M阳性患者中使用奥希替尼+雷莫芦单抗实现了11.0个月（95%CI: 5.5-19.3）的mPFS，并没有明显高于AURA3研究^[58]^中奥希替尼单药实现的10.1个月（95%CI: 8.3-12.3）。在Zhou等^[59]^进行的单臂回顾性研究中奥希替尼+安罗替尼实现了15.5个月（95%CI: 6.19-24.81）的mPFS，以及23.8个月（95%CI: 17.67-29.93）的mOS，接近AURA3研究^[60]^的26.8个月（95%CI: 23.5-31.5）的mOS。

关于奥希替尼和贝伐珠单抗联合作为*EGFR*突变晚期NSCLC一线治疗的研究也逐渐公布结果。Yu等^[61]^开展的单臂Ⅰ期/Ⅱ期研究得到了19个月的mPFS。从数值上来看并没有显著优于FLAURA研究^[13]^中奥西替尼单药的mPFS（18.9个月），ORR（80% *vs* 80%）也没有明显差异。但在有中枢神经系统病灶的患者中，中枢神经系统病灶的ORR达到100%（6/6），优于FLAURA研究^[13]^中的66%，但由于纳入的脑转移患者仅有6例，应谨慎解读这一结果。同样，WJOG9717L研究^[62]^表明奥希替尼联合贝伐珠单抗相较于奥希替尼单药在mPFS方面无法取得额外获益（22.1个月 *vs* 20.2个月，HR=0.862，95%CI：0.531-1.397，*P*=0.213）。

### 4.2 安全性

与第一代EGFR-TKI的A+T模式相似，尽管联合用药的AEs发生率更高，但大多数AEs通常是轻度（1级/2级）、可接受的^[55]^。BOOSTER研究^[56]^中，联合治疗组和单药组出现任何级别AEs的患者比例分别为96%和87%，而3级及以上AEs的患者比例分别为47%和18%。A+T组蛋白尿、高血压的发生率明显升高。

奥希替尼的治疗时间在两组间无显著差异。28%的A+T组患者因AE停用贝伐珠单抗，但事后分析表明，这类停药事件并不影响A+T治疗的PFS^[55]^。

### 4.3 亚组分析

Ex21L858R患者^[56]^和脑转移患者^[55]^在A+T模式的PFS HR仍然相对较低。在BOOSTER试验的亚组分析中，A+T模式可能使有吸烟史患者PFS显著获益（HR=0.57, 95%CI: 0.33-0.98, *P*=0.043），OS也有延长的趋势（HR=0.64, 95%CI: 0.33-1.22, *P*=0.18）^[56]^。这是奥希替尼+贝伐珠单抗取得的少见的阳性结果。在WJOG8715L、WJOG9717L研究^[55, 62]^的亚组分析中也显示A+T模式更适合有吸烟史的患者的趋势。由于伴随突变（尤其是*TP53*共突变）的发生率与吸烟史显著相关^[63]^，有吸烟史的患者疗效更佳的原因可能是该群体中存在伴随突变患者的比例更高。

此外，既往抗血管生成药物治疗史可能会影响A+T模式的疗效^[55]^，这可能是因为长时间使用贝伐珠单抗会减少肿瘤血管的数目而影响药物灌注^[21]^。

## 5 小结与展望

一线治疗*EGFR*突变晚期NSCLC时，第一代EGFR-TKI联合抗血管生成药物相较于单纯第一代EGFR-TKI可以显著提高PFS，然而这种PFS的获益似乎不能转化为OS获益。这可能是因为后续治疗（如奥希替尼）很强的效果掩盖了一线治疗中A+T模式的OS获益，也可能是A+T模式更高的AEs发生率影响了进行后续治疗的患者比例及方案。Ma等^[64]^通过对NEJ026、RELAY等研究进行合并分析，发现A+T组进行后续系统性治疗的比例低于EGFR-TKI单药组（67.9% *vs* 75.3%，合并RR=0.881，95%CI：0.808-0.960，*P*=0.002），进行后续奥希替尼的比例也更低（22% *vs* 27.2%，合并RR=0.858，95%CI：0.642-1.146，*P*=0.299）。这可能是最终OS没有获益的重要原因。同时NEJ026研究和CTONG1509研究使用量表及问卷，发现A+T组的生活质量下降幅度与EGFR-TKI单药组患者没有显著统计学差异，但有更差的趋势^[28, 29]^。这可能是造成上述A+T组患者接受后续治疗比例更低的潜在原因。

奥希替尼联合贝伐珠单抗相较于奥希替尼单药治疗第一/二代EGRF-TKI耐药、T790M突变患者在PFS和OS上都没有取得显著获益。这可能是因为奥希替尼提高PFS和OS的程度非常高，使得联用贝伐珠单抗的额外获益不能突破统计学的显著性边界。奥希替尼联合贝伐珠单抗对比奥希替尼单药一线治疗*EGFR*突变NSCLC的Ⅲ期临床试验NCT04181060正在进行中。

以第一代EGFR-TKI为基础的A+T模式进行的一线治疗可以取得相当高的PFS，甚至能与奥希替尼相媲美。尽管A+T模式是否引起进展患者中T790M突变比例下降仍然存在争议，目前A+T方案耐药后根据耐药机制选择性进行奥希替尼治疗仍是一种值得推荐的模式，尤其是对于*TP53*共突变和脑转移患者，对Ex21L858R患者可能也有较大优势。一项探索厄洛替尼+雷莫芦单抗、后续根据T790M突变情况决定二线使用奥希替尼单药，对比奥希替尼单药一线治疗Ex21L858R患者的随机对照试验正在进行中，以确定晚期Ex21L858R患者的最佳治疗方案^[65]^。

在亚组分析中，部分研究得到A+T模式更适合*TP53*共突变、脑转移患者和Ex21L858R突变的结论。但是应注意这些分析都是基于亚组之间HR的比较和mPFS数值上的大小趋势，并没有明确的统计学验证，甚至有研究存在相反的意见。因此对这些亚组分析的结果的解读应该相当谨慎。*TP53*共突变可以从A+T模式获益可能是其中相对明确和有前景的结论，由于吸烟史和*TP53*共突变的发生存在相关关系，对于存在吸烟史患者的治疗也可以考虑A+T模式。此外，A+T模式在Ex21L858R患者中的更大获益可能也与其阻断了Ex21L858R与TP53等伴随突变的相互作用有关^[66]^。*TP53*突变作为潜在的EGFR-TKI耐药因素，可能是继续探索A+T模式的最佳切入点。

除了≥3级的腹泻外，加入抗血管生成药物并没有显著增加EGFR-TKI相关的AEs，但引入了其特有的高血压、蛋白尿和出血事件。尽管这些AEs大多是可以接受和控制的，但要注意在原有高血压、肾脏疾病和凝血功能障碍的患者中应慎重或避免使用A+T模式。

抗血管生成药物加入EGFR-TKI治疗的时机也值得思考。体外实验发现长时间抗血管治疗在后期可能会减少肿瘤内血管的数量，因此抗血管生成药物可能仅在一定时间内增加肿瘤的药物灌注。部分回顾性研究^[67, 68]^证明在原有EGFR-TKI耐药后进行A+T的再挑战可以继续获益。EGFR-TKI耐药后再加用抗血管生成药物是否可以取得与A+T模式同样的获益，还需要更多的数据来验证。

## References

[1] Huang Z, Su W, Lu T (2020). First-line immune-checkpoint inhibitors in non-small cell lung cancer: current landscape and future progress. Front Pharmacol.

[2] Gelatti ACZ, Drilon A, Santini FC (2019). Optimizing the sequencing of tyrosine kinase inhibitors (TKIs) in epidermal growth factor receptor (*EGFR*) mutation-positive non-small cell lung cancer (NSCLC). Lung Cancer.

[3] Osmani L, Askin F, Gabrielson E (2018). Current WHO guidelines and the critical role of immunohistochemical markers in the subclassification of non-small cell lung carcinoma (NSCLC): Moving from targeted therapy to immunotherapy. Semin Cancer Biol.

[4] Hsu WH, Yang JC, Mok TS (2018). Overview of current systemic management of *EGFR*-mutant NSCLC. Ann Oncol.

[5] Gazdar AF (2009). Activating and resistance mutations of *EGFR* in non-small-cell lung cancer: role in clinical response to EGFR tyrosine kinase inhibitors. Oncogene.

[6] Herbst RS, Morgensztern D, Boshoff C (2018). The biology and management of non-small cell lung cancer. Nature.

[7] Girard N (2018). Optimizing outcomes in *EGFR* mutation-positive NSCLC: which tyrosine kinase inhibitor and when?. Future Oncol.

[8] Noronha V, Patil VM, Joshi A (2020). Gefitinib versus gefitinib plus pemetrexed and carboplatin chemotherapy in *EGFR*-mutated lung cancer. J Clin Oncol.

[9] Yang JJ, Zhou Q, Yan HH (2017). A phase Ⅲ randomised controlled trial of erlotinib *vs* gefitinib in advanced non-small cell lung cancer with *EGFR* mutations. Br J Cancer.

[10] Hosomi Y, Morita S, Sugawara S (2020). Gefitinib alone versus gefitinib plus chemotherapy for non-small-cell lung cancer with mutated epidermal growth factor receptor: NEJ009 study. J Clin Oncol.

[11] Zhang L, Luo Y, Chen J (2021). Efficacy and safety of afatinib in the treatment of advanced non-small-cell lung cancer with *EGFR* mutations: a *meta*-analysis of real-world evidence. J Oncol.

[12] Mok TS, Cheng Y, Zhou X (2018). Improvement in overall survival in a randomized study that compared dacomitinib with gefitinib in patients with advanced non-small-cell lung cancer and *EGFR*-activating mutations. J Clin Oncol.

[13] Soria JC, Ohe Y, Vansteenkiste J (2018). Osimertinib in untreated *EGFR*-mutated advanced non-small-cell lung cancer. N Engl J Med.

[14] Ramalingam SS, Vansteenkiste J, Planchard D (2020). Overall survival with osimertinib in untreated, *EGFR*-mutated advanced NSCLC. N Engl J Med.

[15] Sandler A, Gray R, Perry MC (2006). Paclitaxel-carboplatin alone or with bevacizumab for non-small-cell lung cancer. N Engl J Med.

[16] Naumov GN, Nilsson MB, Cascone T (2009). Combined vascular endothelial growth factor receptor and epidermal growth factor receptor (EGFR) blockade inhibits tumor growth in xenograft models of EGFR inhibitor resistance. Clin Cancer Res.

[17] Larsen AK, Ouaret D, El Ouadrani K (2011). Targeting EGFR and VEGF(R) pathway cross-talk in tumor survival and angiogenesis. Pharmacol Therapeutics.

[18] De Luca A, Carotenuto A, Rachiglio A (2008). The role of the EGFR signaling in tumor microenvironment. J Cell Physiol.

[19] Perdrizet K, Leighl NB (2019). The role of angiogenesis inhibitors in the era of immune checkpoint inhibitors and targeted therapy in metastatic non-small cell lung cancer. Curr Treat Options Oncol.

[20] Bozec A, Sudaka A, Fischel JL (2008). Combined effects of bevacizumab with erlotinib and irradiation: a preclinical study on a head and neck cancer orthotopic model. Br J Cancer.

[21] Dickson PV, Hamner JB, Sims TL (2007). Bevacizumab-induced transient remodeling of the vasculature in neuroblastoma xenografts results in improved delivery and efficacy of systemically administered chemotherapy. Clin Cancer Res.

[22] Li H, Takayama K, Wang S (2014). Addition of bevacizumab enhances antitumor activity of erlotinib against non-small cell lung cancer xenografts depending on VEGF expression. Cancer Chemother Pharmacol.

[23] Feng PH, Chen KY, Huang YC (2018). Bevacizumab reduces S100A9-positive MDSCs linked to intracranial control in patients with *EGFR*-mutant lung adenocarcinoma. J Thorac Oncol.

[24] Herbst RS, Ansari R, Bustin F (2011). Efficacy of bevacizumab plus erlotinib versus erlotinib alone in advanced non-small-cell lung cancer after failure of standard first-line chemotherapy (BeTa): a double-blind, placebo-controlled, phase 3 trial. Lancet.

[25] Seto T, Kato T, Nishio M (2014). Erlotinib alone or with bevacizumab as first-line therapy in patients with advanced non-squamous non-small-cell lung cancer harbouring *EGFR* mutations (JO25567): an open-label, randomised, multicentre, phase 2 study. Lancet Oncol.

[26] Saito H, Fukuhara T, Furuya N (2019). Erlotinib plus bevacizumab versus erlotinib alone in patients with *EGFR*-positive advanced non-squamous non-small-cell lung cancer (NEJ026): interim analysis of an open-label, randomised, multicentre, phase 3 trial. Lancet Oncol.

[27] Yamamoto N, Seto T, Nishio M (2021). Erlotinib plus bevacizumab *vs* erlotinib monotherapy as first-line treatment for advanced *EGFR* mutation-positive non-squamous non-small-cell lung cancer: Survival follow-up results of the randomized JO25567 study. Lung Cancer.

[28] Kawashima Y, Fukuhara T, Saito H (2022). Bevacizumab plus erlotinib versus erlotinib alone in Japanese patients with advanced, metastatic, *EGFR*-mutant non-small-cell lung cancer (NEJ026): overall survival analysis of an open-label, randomised, multicentre, phase 3 trial. Lancet Respir Med.

[29] Zhou Q, Xu CR, Cheng Y (2021). Bevacizumab plus erlotinib in Chinese patients with untreated, *EGFR*-mutated, advanced NSCLC (ARTEMIS-CTONG1509): A multicenter phase 3 study. Cancer Cell.

[30] Nakagawa K, Garon EB, Seto T (2019). Ramucirumab plus erlotinib in patients with untreated, *EGFR*-mutated, advanced non-small-cell lung cancer (RELAY): a randomised, double-blind, placebo-controlled, phase 3 trial. Lancet Oncol.

[31] Zhao H, Yao W, Min X (2021). Apatinib plus gefitinib as first-line treatment in advanced *EGFR*-mutant NSCLC: The phase Ⅲ ACTIVE study (CTONG1706). J Thorac Oncol.

[32] Lu S, Zhou JY, Niu XM (2021). Fruquintinib with gefitinib as first-line therapy in patients carrying *EGFR* mutations with advanced non-small cell lung cancer: a single-arm, phase Ⅱ study. Transl Lung Cancer Res.

[33] Jiang Z, Zhang J, Sun H (2021). Icotinib alone or with bevacizumab as first-line therapy in Chinese patients with advanced nonsquamous non-small cell lung cancer and activating *EGFR* mutations: A retrospective study. Thorac Cancer.

[34] Ohmori T, Yamaoka T, Ando K (2021). Molecular and clinical features of EGFR-TKI-associated lung injury. Int J Mol Sci.

[35] Gressett SM, Shah SR (2009). Intricacies of bevacizumab-induced toxicities and their management. Ann Pharmacother.

[36] Chen Y, Wen S, Wu Y (2021). Efficacy and safety of first-generation epidermal growth factor receptor (EGFR) tyrosine kinase inhibitors (TKIs) combined with chemotherapy or antiangiogenic therapy as first-line treatment in patients with *EGFR*-mutant non-small cell lung cancer: A systematic review and *meta*-analysis. Crit Rev Oncol Hematol.

[37] Nakagawa K, Garon EB, Seto T (2019). Ramucirumab plus erlotinib in patients with untreated, *EGFR*-mutated, advanced non-small-cell lung cancer (RELAY): a randomised, double-blind, placebo-controlled, phase 3 trial. Lancet Oncol.

[38] Hamada S, Ichiyasu H, Ikeda T (2019). Protective effect of bevacizumab on chemotherapy-related acute exacerbation of interstitial lung disease in patients with advanced non-squamous non-small cell lung cancer. BMC Pulm Med.

[39] Skoulidis F, Heymach JV (2019). Co-occurring genomic alterations in non-small-cell lung cancer biology and therapy. Nat Rev Cancer.

[40] Wheler JJ, Janku F, Naing A (2016). TP53 alterations correlate with response to VEGF/VEGFR inhibitors: implications for targeted therapeutics. Mol Cancer Ther.

[41] Said R, Hong DS, Warneke CL (2013). *P53* mutations in advanced cancers: clinical characteristics, outcomes, and correlation between progression-free survival and bevacizumab-containing therapy. Oncotarget.

[42] Zeng L, Xiao L, Jiang W (2020). Investigation of efficacy and acquired resistance for EGFR-TKI plus bevacizumab as first-line treatment in patients with *EGFR* sensitive mutant non-small cell lung cancer in a Real world population. Lung Cancer.

[43] Nakagawa K, Nadal E, Garon EB (2021). RELAY subgroup analyses by *EGFR* Ex19del and Ex21L858R mutations for ramucirumab plus erlotinib in metastatic non-small cell lung cancer. Clin Cancer Res.

[44] Zhong D, Zhang C, Zhang Y (2020). Anlotinib combined with icotinib provides a promising first-line treatment option for *EGFR* positive NSCLC patients harboring concomitant mutations: Exploratory analysis of the ALTER-L004 study. Ann Oncol.

[45] Page S, Milner-Watts C, Perna M (2020). Systemic treatment of brain metastases in non-small cell lung cancer. Eur J Cancer.

[46] Wang H, Xing R, Li M (2021). P48.08 The efficacy and clinical survival outcome of different first-line treatments in *EGFR* mutant non-small cell lung cancer with brain metastases. J Thorac Oncol.

[47] Besse B, Lasserre SF, Compton P (2010). Bevacizumab safety in patients with central nervous system metastases. Clin Cancer Res.

[48] Zhu JQ, Zhong WZ, Zhang GC (2008). Better survival with *EGFR* exon 19 than exon 21 mutations in gefitinib-treated non-small cell lung cancer patients is due to differential inhibition of downstream signals. Cancer Lett.

[49] Liu Y, Ren Z, Wang J (2016). Epidermal growth factor receptor-tyrosine kinase inhibitor therapy is especially beneficial to patients with exon 19 deletion compared with exon 21 L858R mutation in non-small-cell lung cancer: Systematic review and *meta* analysis. Thorac Cancer.

[50] Piccirillo MC, Bonanno L, Garassino MC (2022). Addition of bevacizumab to erlotinib as first-line treatment of patients with *EGFR*-mutated advanced nonsquamous non-small cell lung cancer. The BEVERLY multicenter randomized phase Ⅲ trial. J Thorac Oncol.

[51] Zhao Y, Cheng B, Chen Z (2021). Toxicity profile of epidermal growth factor receptor tyrosine kinase inhibitors for patients with lung cancer: A systematic review and network *meta*-analysis. Crit Rev Oncol Hematol.

[52] Kuo CS, Chiu TH, Tung PH (2022). Afatinib treatment alone or with bevacizumab in a real-world cohort of non-small cell lung cancer patients with epidermal growth factor receptor mutation. Cancers (Basel).

[53] Ko R, Shukuya T, Imamura CK (2021). Phase I study of afatinib plus bevacizumab in patients with advanced non-squamous non-small cell lung cancer harboring *EGFR* mutations. Transl Lung Cancer Res.

[54] Hata A, Katakami N, Kaji R (2018). Afatinib plus bevacizumab combination after acquired resistance to EGFR tyrosine kinase inhibitors in *EGFR*-mutant non-small cell lung cancer: Multicenter, single-arm, phase 2 trial (ABC Study). Cancer.

[55] Akamatsu H, Toi Y, Hayashi H (2021). Efficacy of osimertinib plus bevacizumab *vs* osimertinib in patients with *EGFR* T790M-mutated non-small cell lung cancer previously treated with epidermal growth factor receptor-tyrosine kinase inhibitor: West Japan Oncology Group 8715L phase 2 randomized clinical trial. JAMA Oncol.

[56] Soo RA, Han JY, Dafni U (2022). A randomised phase Ⅱ study of osimertinib and bevacizumab versus osimertinib alone as second-line targeted treatment in advanced NSCLC with confirmed *EGFR* and acquired T790M mutations: the European Thoracic Oncology Platform (ETOP 10-16) BOOSTER trial. Ann Oncol.

[57] Yu HA, Paz-Ares LG, Yang JC (2021). Phase I study of the efficacy and safety of ramucirumab in combination with osimertinib in advanced T790M-positive *EGFR*-mutant non-small cell lung cancer. Clin Cancer Res.

[58] Mok TS, Wu YL, Ahn MJ (2017). Osimertinib or platinum-pemetrexed in *EGFR* T790M-positive lung cancer. N Engl J Med.

[59] Zhou B, Gong Q, Li B (2022). Clinical outcomes and safety of osimertinib plus anlotinib for patients with previously treated *EGFR* T790M-positive NSCLC: A retrospective study. J Clin Pharm Ther.

[60] Papadimitrakopoulou VA, Mok TS, Han JY (2020). Osimertinib versus platinum-pemetrexed for patients with EGFR T790M advanced NSCLC and progression on a prior EGFR-tyrosine kinase inhibitor: AURA3 overall survival analysis. Ann Oncol.

[61] Yu HA, Schoenfeld AJ, Makhnin A (2020). Effect of osimertinib and bevacizumab on progression-free survival for patients with metastatic *EGFR*-mutant lung cancers: A phase 1/2 single-group open-label trial. JAMA Oncol.

[62] Kenmotsu H, Wakuda K, Mori K (2022). Randomized phase 2 study of osimertinib plus bevacizumab versus osimertinib for untreated patients with nonsquamous NSCLC harboring *EGFR* mutations: WJOG9717L study. J Thorac Oncol.

[63] Chen H, Liu M, Dai Z (2020). Concomitant genetic alterations are associated with response to EGFR targeted therapy in patients with lung adenocarcinoma. Transl Lung Cancer Res.

[64] Ma JT, Guo YJ, Song J (2021). Rational application of first-line EGFR-TKIs combined with antiangiogenic inhibitors in advanced *EGFR*-mutant non-small-cell lung cancer: a systematic review and *meta*-analysis. Biomed Res Int.

[65] Haratake N, Hayashi H, Shimokawa M (2022). Phase Ⅲ clinical trial for the combination of erlotinib plus ramucirumab compared with osimertinib in previously untreated advanced or recurrent non-small cell lung cancer positive for the L858R mutation of *EGFR*: REVOL858R (WJOG14420L). Clin Lung Cancer.

[66] Hellyer JA, White MN, Gardner RM (2022). Impact of tumor suppressor gene co-mutations on differential response to EGFR TKI therapy in *EGFR* L858R and exon 19 deletion lung cancer. Clin Lung Cancer.

[67] Zhang C, Cao H, Cui Y (2021). Concurrent use of anlotinib overcomes acquired resistance to EGFR-TKI in patients with advanced *EGFR*-mutant non-small cell lung cancer. Thorac Cancer.

[68] Yang X, Xia Y, Xu L (2021). Efficacy and safety of combination treatment with apatinib and osimertinib after osimertinib resistance in epidermal growth factor receptor-mutant non-small cell lung carcinoma - A retrospective analysis of a multicenter clinical study. Front Mol Biosci.

